# Forensic Case Formulation: Exploring the Knowledge, Opinions, and Training Experiences of Staff Working Within the Offender Personality Disorder Pathway

**DOI:** 10.1177/0306624X231219986

**Published:** 2024-01-28

**Authors:** Victoria Wheable, Jason Davies

**Affiliations:** 1 Swansea University, UK

**Keywords:** forensic case formulation, offender personality disorder pathway, staff training, forensic mental health

## Abstract

Forensic case formulation (FCF) is a key activity within the Offender Personality Disorder Pathway (OPDP), performed by OPDP specialist offender managers (OMs) and psychologists. Although FCF training is provided to OMs, there are a number of questions about the adequacy and effectiveness of this training. Furthermore, it is unclear whether psychologists receive sufficient support to keep their FCF skills relevant and effective over time. This study aimed to investigate the FCF training experiences of OPDP staff, to assess staff satisfaction with this training, to identify ways of improving this training, and to explore the value of FCF from a staff perspective. To meet these aims, OPDP staff were asked to complete an online Qualtrics survey disseminated nationally. Results reveal a lack of standardized FCF training across the OPDP, contributing to poor staff satisfaction and confidence. These results highlight a need for FCF training improvement within the OPDP.

## Introduction

The Offender Personality Disorder Pathway (OPDP) was co-commissioned in 2011 by the National Offender Management Service (NOMS) and the National Health Service (NHS) to better manage and treat high-risk offenders with traits of a personality disorder (Joseph & Benefield, 2012). The main aims of the OPDP are to reduce reoffending rates, to improve the psychological health of offenders, and to develop the confidence, competence, and skills of staff working with these offenders (National Offender Management Service [NOMS] & NHS England, 2015a). Although reductions in offending behavior as a result of the OPDP have not yet been statistically proven, qualitative research has identified positive impacts for both offenders (psychological health, trust, and safety) and staff (safety, competence, and confidence; HMPPS, 2022; Wheable & Davies, 2020).

Once an offender has been screened into the OPDP, they receive tailored support often shaped on the basis of an individualized case formulation. Case formulation can be described as a “hypothesis about the causes, precipitants and maintaining influences of a person’s psychological, interpersonal, and behavioural issues” (Eells, 2007, p.4) and is used to “organise information, guide treatment and measure change” (Davies et al., 2013, p. 305).

Within the OPDP, forensic case formulation (FCF) is used to gain a psychological understanding of each offender’s criminal behavior, clinical problems, and criminogenic needs (Joseph & Benefield, 2012). Each formulation generally consists of: an overview of the case, a hypothesized psychological explanation of the causes, development, and maintaining influences of offender’s presenting problems, and a set of recommendations to be utilized by the OM to facilitate further progress within the case and/or reduce the offender’s risk of harm. These case formulations are often informed by psychological research (e.g., trauma; Webermann et al, 2020) or psychotherapeutic models (e.g., schema therapy; Beckley, 2022). Whilst forensic case formulation is not a risk assessment or needs assessment method per se, theoretically anchored explanations are commonly used to provide an understanding of behavior such as violence toward others.

Three different “levels” of FCFs are written within the OPDP, to provide “flexibility in response to widely divergent contexts and practitioner needs” (NOMS & NHS England, 2015b, p. 40). Level 1 FCFs are the simplest (<1 page in length), whereas level 3 FCFs are most complex (>2 pages in length). Specialist offender managers (OMs) within the OPDP are required to take an active role in writing lower-level FCFs alongside psychologists (NOMS & NHS England, 2015b). However, as OMs often have little or no psychological training, there has been scepticism regarding this strategy (i.e., Brown & Völlm, 2013, 2016). This is because the consequences of producing *inaccurate* formulations within forensic services could be “very great indeed” (Hart et al., 2011, p. 122), including the possibility of “additional adverse outcomes such as repeat serious offending, significant injuries and trauma to others, and large costs of incarceration and long-term treatment” (Sturmey & McMurran, 2011, p. 288). This is concerning, particularly in the context of managing and treating high-risk offenders within the OPDP, and thus highlights the importance of ensuring that all those who write case formulations have the necessary skills to do so competently.

Several studies have examined the baseline FCF skills of OMs and whether these can be improved with training (Brown et al., 2016; Mapplebeck et al., 2017; Minoudis et al., 2013; Radcliffe et al., 2018). These studies have reported mixed results, but the majority of their findings indicate that such training *can* improve the FCF skills of OMs, meaning that this training is important for OMs to receive. Nevertheless, it is not currently known how much FCF training OMs within the OPDP receive, whether they feel satisfied with the quantity and quality of this training, and whether they feel confident in their skills after receiving this training.

Furthermore, although psychologists are responsible for writing higher-level FCFs, no research to date has explored *if* or *how* the FCF skills of psychologists are kept relevant and effective over time. The importance of this issue has been demonstrated by Hopton et al. (2018), who used the Case Formulation Quality Checklist—Revised (McMurran & Bruford, 2016) to rate a sample of 121 risk formulations produced by psychologists within forensic inpatient settings and found them to be of generally poor to intermediate quality.

With these issues in mind, the current study aimed to (a) investigate the quantity and quality of FCF training provided to OPDP staff (including OMs and psychologists), (b) to identify how FCF training could be best improved, and (c) to understand the general utility and value of FCF from an OPDP staff perspective. OPDP staff were asked to complete a 20-minute online survey in which they detailed their FCF training experiences and opinions and provided suggestions for training improvement. Results of this research will establish the adequacy of current FCF training provisions within the OPDP, and create opportunity for improving the utility and value of this training.

## Method

### Participants

All OPDP staff members responsible for writing FCFs were invited to take part in the online survey. To recruit these participants, OPDP co-commissioners distributed the survey link to OPDP team leaders and requested them to circulate it to all eligible staff.

### Materials

The online survey was developed in Qualtrics. It contained a total of 46 demographic, multiple-choice, and open-ended questions.

After providing demographic information, participants reported the number of hours of FCF training they had received, their level of satisfaction with the quantity and quality of this training (from 1 “Very Unsatisfied” to 4 “Very Satisfied”), how often their FCF skills were assessed (from 1 “Never” to 7 “Weekly”), and how confident they felt in their FCF skills (from 1 “Very Unconfident” to 4 “Very Confident”). Participants were also asked to describe any experiences of providing FCF training to others. Next, participants were asked to provide qualitative suggestions for how FCF training within the OPDP could be improved, and to provide their opinions on the general utility and value of FCF. Results from the latter question were expected to indicate the receptivity of OPDP staff to any future FCF training developed, and also to facilitate understanding of what OPDP staff perceive the main outcomes of FCF to be (i.e., if they believe FCF is useful and effective, *what* is it most useful and effective at doing?).

### Procedure

Approval was first obtained from HMPPS National Research Committee (ref. 2018-089) and Swansea University Research Ethics Committee (ref. 0240).

Eligible staff were e-mailed the link to the online survey via their OPDP team leaders. After reading the electronic information sheet, participants were required to select “Yes, I Consent” before moving ahead to the survey. If they selected “No, I Do Not Consent,” they were taken to the end of the survey and thanked for their time. After 3 months, a reminder e-mail was sent to staff members who had not yet completed the survey. The survey remained active for 6 months between August 2018 and February 2019.

### Data Analysis

Responses to multiple-choice questions were examined using frequency counts in IBM SPSS Statistics. Responses to open questions were examined with inductive thematic analysis using guidance by Braun and Clarke (2006).

## Results

### Survey Respondents

A total of 55 OPDP staff completed the online survey. Table 1 contains the demographic information of these participants. Although the response rate to the survey could not be reliably calculated (due to no central record of the number of staff employed by the OPDP who write FCFs), the demographic information indicates that a relatively diverse range of OPDP staff participated.

**Table 1.** Demographic Information of Survey Respondents.

Demographic variable	Count
Sex	
Male	9
Female	46
Age (years)	
25–30	8
31–40	33
41–50	7
>50	7
Years working within profession (years)	
<5	8
5–10	17
11–15	15
>15	15
Years working within OPDP (years)	
<1	11
1–2	10
2–5	23
>5	11
Job title	
Psychologist	26
Assistant psychologist	9
OM	11
Health practitioner	4
Therapist	4
Service manager	1

### Experience of Writing FCFs

Participants reported having written between 1 and 1,200 formulations (Mdn = 70). Health practitioners had written the most (Mdn = 400), whereas assistant psychologists had written the fewest (Mdn = 30). This is likely due to assistant psychologists being in post for a comparatively smaller number of years. Over 90% of participants reported being responsible for writing Level 2 FCFs (this included the majority of the OMs). Level 3 FCFs were most typically written by psychologists.

### Formulation Training Experience

#### Sources and Methods of Formulation Training

Participants had received formulation training from two different sources on average (ranging from one to five sources overall). All participants had received formulation training as part of a professional training program (*n* = 55). A smaller number (*n* = 23) reported having receiving formulation training as part of their OPDP job induction. All but two of the participating psychologists had also received formulation training as part of their doctoral program. The most common methods used to deliver this formulation training had been classroom-style lectures (84%), case vignettes (81%), and group tasks (69%).

#### Frequency of Formulation Training

Just over half of participants (*n* = 28) had received formulation training within the past year. In contrast, three participants reported not having received formulation training in over 5 years, and four participants reported *never* having received formulation training. Assistant psychologists were most likely to have received formulation training within the past year (78%), whereas psychologists were the least likely^1^ (31%; see Table 2).

**Table 2.** Time of Last Formulation Training and Median Hours of Formulation Training Ever Received (Split by Job Role).

Job role	Time since last formulation training (*n* and %) and amount of training ever received (Mdn)					
	Never trained	>5 years ago	2–5 years ago	1–2 years ago	<1 year ago	Overall training hours
Psychologist	1 (4%)	2 (8%)	9 (35%)	6 (23%)	8 (31%)	21.5
Assistant psychologist	0 (0%)	0 (0%)	0 (0%)	2 (22%)	7 (78%)	10
Offender manager	2 (18%)	0 (0%)	1 (9%)	1 (9%)	7 (64%)	10
Health practitioner	0 (0%)	1 (25%)	0 (0%)	0 (0%)	3 (75%)	16.5
Therapist	0 (0%)	0 (0%)	1 (25%)	0 (0%)	3 (75%)	25
Service manager	1 (100%)	0 (0%)	0 (0%)	0 (0%)	0 (0%)	0

### Quantity, Quality, and Adequacy of Formulation Training

Participants had received between 0 and 100 hours of formulation training in total (Mdn = 15 hours). The right-hand column of Table 2 shows that therapists had received the most training (Mdn = 25 hours), whereas the service manager had received the least (0 hours). Those responsible for writing *only* level 1 FCFs had received the least amount of training (Mdn = 0 hours), whereas those writing level 3 FCFs had received the most (Mdn = 20 hours).

Only 27% of participants reported being “Very Satisfied” with the amount of training they had received. Those who were at least “Somewhat Satisfied” had received more training (Mdn = 20 hours) than those who were unsatisfied (Mdn = 5 hours). Only 41% of those who had received formulation training reported that they were “Very Satisfied” with the *quality* of this training.

**Figure 1.**
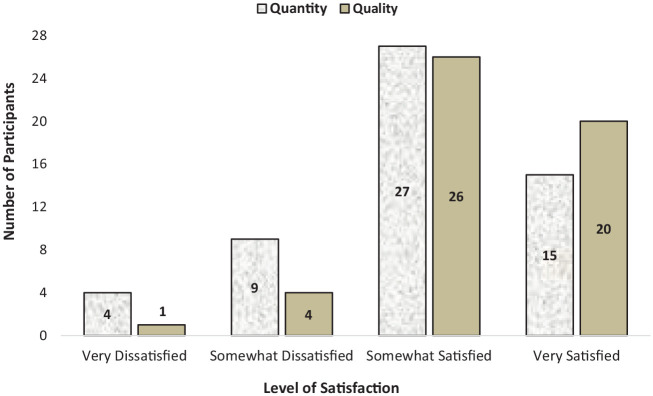
Participant satisfaction with quantity and quality of formulation training. *Note.* Participants who had never received FCF training (*n* = 4) provided “quantity” ratings, but not “quality” ratings. Two of these participants were “Very Dissatisfied” with quantity.

In sum, only 20% of participants were “Very Satisfied” with *both* the quantity and quality of formulation training received. Figure 1 provides a full overview of these results.

### Formulation Assessment

**Figure 2.**
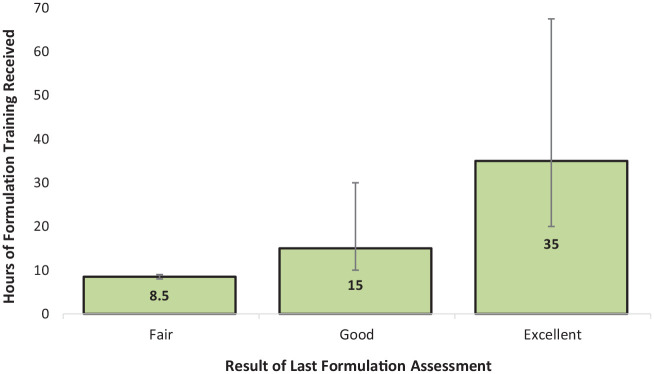
Median hours of formulation training received by participants who received fair, good, or excellent feedback during their last formulation assessment. *Note.* Error bars represent interquartile range.

38% of participants stated that their FCF skills were assessed at least monthly (i.e., within supervision). However, 16% of participants (*n* = 9) reported that their skills had *never* been assessed. Of those that had been assessed, only 20% reported receiving “excellent” feedback at their last assessment. However, no participant reported that the result of their last assessment highlighted a need for improvement. Please see Table 3 for a summary of this information split by job role. Participants who had received “excellent” feedback at their last FCF assessment had received more formulation training (Figure 2).

**Table 3.** Result of Last Formulation Assessment as Reported by Participants (Split by Job Role).

Job Role	Outcome of last formulation assessment (*n* and %) and median hours of formulation training received				
	Never assessed	Need for improvement	Fair	Good	Excellent
Psychologist	5 (19%)	0 (0%)	1 (4%)	15 (58%)	5 (19%)
Assistant psychologist	0 (0%)	0 (0%)	1 (11%)	7 (78%)	1 (11%)
Offender manager	3 (27%)	0 (0%)	0 (0%)	6 (55%)	2 (18%)
Health practitioner	0 (0%)	0 (0%)	0 (0%)	3 (75%)	1 (25%)
Therapist	0 (0%)	0 (0%)	0 (0%)	4 (100%)	0 (0%)
Service manager	1 (100%)	0 (0%)	0 (0%)	0 (0%)	0 (0%)

### Confidence in Formulation Skills

**Figure 3.**
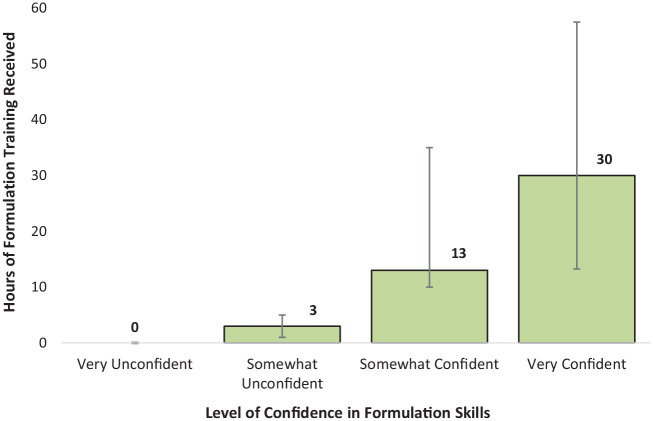
Median hours of formulation training received by participants with different levels of confidence in their formulation skills.

Only 22% of participants reported feeling “Very Confident” in their FCF skills, with one participant feeling “Very Unconfident”. Table 4 shows that confidence was not a simple product of job role. However, those with most confidence had received most FCF training (Figure 3).

**Table 4.** Confidence in Formulation Skills as Reported by Participants (Split by Job Role).

Job role	Confidence in formulation skills (%)			
	Very unconfident	Somewhat unconfident	Somewhat confident	Very confident
Psychologist	0 (0)	1 (4)	16 (61)	9 (35)
Assistant psychologist	0 (0)	0 (0)	7 (78)	2 (22)
Offender manager	1 (9)	1 (9)	8 (73)	1 (9)
Practitioner	0 (0)	1 (25)	3 (75)	0 (0)
Therapist	0 (0)	0 (0)	4 (100)	0 (0)
Service manager	0 (0)	0 (0)	1 (100)	0 (0)

Level of confidence was also found to correspond with the *quality* of formulation training received; 40% of those who were “Very Satisfied” with the quality of their training also reporting feeling “Very Confident” in their formulation skills. *None* of those who were “Somewhat Dissatisfied” or “Very Dissatisfied” with the quality of their training were “Very Confident” in their skills.

### Providing Training to Others

More than half of participants (*n* = 30) had delivered formulation training to others. This commonly consisted of providing informal “on the job” assistance (83%), providing semi-formal training during supervision (73%), and/or providing formal training as part of an official program (53%). The recipients of this training were most commonly reported to be OMs (20%), psychologists working outside of the OPDP (20%), and/or healthcare staff (17%). Psychologists were those most likely to have provided training to others.

Only 33% of participants who had provided formulation training to others (10 of 30) reported feeling “Very Confident” in their formulation skills. This suggests that some OPDP staff are required or have found it necessary to train others to write formulations without first being fully confident in their own skills.

When these participants were asked *how* they had acquired the skills to deliver formulation training, 14 of these 30 participants reported that they had not received any guidance at all. However, of those who had not received any guidance, over half (*n* = 8) stated this was due to being a recognized expert within the formulation field. However, only five of these eight “experts” reported feeling “Very Confident” in their own formulation skills.

### Suggestions for Training Improvement

All 55 participants provided at least one suggestion for improving OPDP formulation training. Four themes emerged from the thematic analysis: “Accessibility of Training,” “Improving Training Methods,” “Improving Training Content,” and “Providing Staff Support.”

#### Accessibility of Training

This theme centered around what staff commonly described as a lack of access to FCF training within the OPDP. Corroborating the multiple-choice responses, some participants stated that although they were required to write FCFs as part of their duties, they had never received FCF training. Some participants reported that they were not aware that FCF training was offered by the OPDP, raising questions about its accessibility. Many of these participants stated that they would find it beneficial to attend formulation training if it were offered:

*I have not been aware that specific formulation training exists. Attending that may*

*have been helpful prior to completing formulations (Participant 5)*

*If they advertised the training on Kahootz* ^2^ *as I have never seen an opportunity to*

*attend case formulation training (P27)*

*I would appreciate formal case formulation training (P35)*

Participants who *had* received FCF training indicated that additional training would be useful. It was commonly suggested that formulation training be an ongoing process rather than a one-off occurrence:

*I think having more of it on a regular basis (P12)*

*I feel like regular ‘refresher’ training should be available. Like an annual event for*

*those who are writing formulations regularly to go to share good practice, explore*

*issues etc (P44)*

*As part of a rolling programme of training. It would be welcomed and useful to have*

*such training (P52)*

*In my opinion - Training should be ongoing (P55)*

#### Improving Training Methods

This theme consisted of participants suggesting ways to improve the *methods* used to deliver FCF training. These suggestions fell into a number of distinct categories which will be discussed as smaller subthemes. A common thread between these subthemes is that any method used within training should maximize the likelihood that any skills developed are relevant and readily transferable to practice.

*Real Case Examples.* The most common method suggested by participants was that training should involve the formulation of *real* cases rather than fictitious case vignettes.

*Ensuring that people can bring real anonymised cases in order to apply the*

*training (P10)*

*Using ‘real’ cases from the caseload of the attendees (P15)*

*Using a real-life case who people on the course are aware of (P17)*

*Use of participants’ real case experiences/material – making it ‘live’ and relevant*

*for practice (P43)*

##### “Hands On” Practice

Participants felt that the methods used within their past training had often been too “passive.” This supports the earlier multiple-choice question responses, where participants reported that the most common method of delivery used within their formulation training had been “classroom-style lectures”. Participants commonly suggested that staff should be given the opportunity to apply their newly learned skills by actively writing “practice” FCFs and receiving feedback on these during training.

*Self-formulation rather than theory/PowerPoint exclusive training (P31)*

*More practical hands-on experience of trying to develop formulations (P42)*

*Being provided with the opportunity to write a case formulation and then provided*

*with feedback on this (P50)*

*Make it as interactive as possible please (P51)*

##### Expert and Benchmark Formulations

A third suggestion for improving training methods was to use examples of “expert” or “gold standard” formulations as a guide.

I would also like it to involve a benchmarking exercise (P12)

Expert demonstration (P30)

Examples of gold standard formulations (P51)

Use of ‘gold standard’ level three examples (P50)

Although it might be beneficial to use such guides within training, it is problematic to assume that “expert” or benchmark FCFs are of a gold standard; Mumma and Mooney (2007), state that “the extent to which expert clinicians’ formulations are a ‘gold standard’ is unclear” (p. 475).

#### Improved Training Content

The third theme identified within the suggestions for training improvement was that the *content* of formulation training could also be improved. Two subthemes were identified within this theme:

##### Clarity and Consistency

Participants indicated that key features of FCF within the OPDP (such as the different *levels* and *types*) should be better clarified within training. This indicates that staff feel unsure about these particular aspects:

*Clear guidelines around what should be included in each level of formulation (P14)*

*I’d like to better understand the differences between the three levels as this seems to*

*vary (P33)*

*More differentiation of the different levels of formulation (1, 2 and 3), and more*

*clarity about how case formulation differs from risk formulation, and how risk*

*formulation differs from problem formulation (P49)*

Potentially highlighting the source of confusion around these aspects, participants stressed that FCF training should be *standardized* to ensure consistent knowledge across teams:

*So everyone was working within the same standards and knew what specific*

*information to put into a formulation (P13)*

*Having a standardised training program would also be helpful to ensure consistency*

*across services within the pathway (P28)*

*Consistent expectations, in practice (not in theory/the book), between OPD teams*

*about what formulations need to contain (P32)*

##### Psychological Content

The second subtheme indicated that there is a need for FCF training to include more information about different *psychological models and theories* and how to incorporate these into FCFs:

*It would be useful to explore additional models (P23)*

*A general better understanding of the different psychological theories which could*

*be used to help hypothesise a problem (P26)*

*It would be useful to receive some training around different theoretical models (P38)*

*Use of different psychological models to formulate (P47)*

This finding is important, as one of the main purposes of FCF within the OPDP is to develop a psychological understanding of the causes and development of each service user’s presenting problems.

#### Providing Staff Support

The final theme identified within the suggestions for training improvement emphasized a need for further support to be provided to less-experienced staff who may otherwise develop negative associations toward FCF.

*It can feel intimidating for people when it’s new to them (P16)*

*I have found that the language we use (even just the term formulation) seems to*

*create anxiety (P6)*

However, participants provided a number of suggestions for how to reduce these potential issues:

*Finding ways to manage this anxiety and helping to increase staff confidence (P6)*

*Methods that allow for all preferences in terms of learning (P10)*

*Drawing on attendees existing strengths in case formulation (even where they might*

*not be aware that they have these!) (P15)*

*Ensuring the language/terminology is accessible to staff of all disciplines (P31)*

These responses indicate that identifying the needs of staff and supporting them both before and during this training process is an essential step that should be prioritized.

### Staff Opinions on the Utility and Effectiveness of Formulation

All 55 participants provided an opinion on whether FCF itself is useful or effective within the OPDP. Five themes emerged from the thematic analysis: “Improving Understanding,” “Facilitating Progress,” “Improving Relationships,” “Main Offender Outcomes,” and “Barriers to Usefulness and Effectiveness.”

#### Improving Understanding

Many participants reported finding FCF to be useful and effective in “improving understanding” for staff and service users. This theme was broken down into two subthemes:

##### Staff Understanding

Participants found FCF particularly useful for improving their own or other staff member’s understanding of an offender’s presenting problems:

*I regularly use it with probation staff to help make sense of offenders’ difficulties*

*and feel it can help improve staff understanding (P6)*

*It helps to make sense of behaviours that can otherwise seem confusing (P33)*

*The most useful way of understanding a person’s actions (P37)*

Building an understanding of an offenders presenting problems is typically one of the main aims of FCF; these comments therefore suggests that this aim is being met well. Participants also indicated that FCF allowed them to better understand each offender as a *person*:

*It gives a space to understand clients at much deeper level (P3)*

*Makes sense of the person as a whole (P32)*

*Formulation goes beyond the person’s problems and puts them in the context of the*

*person him or herself (P49)*

By facilitating this deeper understanding, some participants also indicated that FCF can enable the development of more staff empathy and compassion toward offenders:

*I feel it can help increase staff understanding and empathy towards the service*

*user (P6)*

*It supports a more thoughtful and compassionate approach (P32)*

*It helps improve understanding of clients, increases empathy and results in more*

*positive and compassionate approaches to working with clients (P47)*

##### Offender Understanding

Participants also reported that FCF is useful and effective in providing offenders with a better understanding of their *own* presenting problems:

*It helps the service user to spend time thinking about some of their behaviours and*

*start to understand themselves a little more (P14)*

*It helps the individual have a greater understanding of their own behaviour (P30)*

*I think that this can often help individuals to better understand themselves, their*

*behaviour and the problems that they are experiencing (P42)*

*For an offender it can be a lightbulb moment that helps them think about how they*

*have arrived in a certain situation (P51)*

Providing offenders with this type of insight is likely to be a valuable function of FCF within the OPDP, as it presents an opportunity for these offenders to identify maladaptive patterns which may then enable them to make steps toward positive change.

#### Improving Relationships

A second way in which staff indicated that FCF is useful and effective within the OPDP is by improving staff-offender relationships:

*Helps the therapeutic relationship (P3)*

*I believe it can improve relationships (P6)*

*It can be helpful in developing a more therapeutic relationship (P21*

*Encourages them to forge relationships with their clients (P51)*

Although it is not known *how* FCF is able to improve these relationships, one possibility is that this occurs via improvements in staff understanding and empathy toward offenders facilitated by the FCF.

#### Facilitating Progress

The third theme identified within participant responses was that FCF is useful and effective at facilitating and guiding further progress within each case. Three different ways of facilitating progress were identified:

*Identifying New Perspectives for Staff.* Staff indicated that FCF facilitates progress by identifying new directions or approaches to take in each case:

*We often see OM’s develop new perspectives of their case (P8)*

*It can really help someone to think outside of the box (P10)*

*Allows us to look at other perspectives (P16)*

By identifying these new perspectives and approaches, FCF may enable staff to move forward with previously “stuck” or difficult cases. This is likely to be an extremely useful function of formulation within the OPDP, as cases screened into the service are often highly complex and challenging to engage.

Participants additionally indicated that by identifying these new perspectives and approaches, staff can feel more confident and supported in the ways in which they work with each offender:

*For the OM’s who choose to engage in the pathway, there seems to be a genuine*

*feeling of being supported and seeking help (P8)*

*The process assists OMs to gain confidence in working with OPD clients (P35)*

*It provides reassurance as to the approach you should use with an offender (P38)*

##### Identifying New Perspectives for Service Users

Participants also indicated that FCF can enable *offenders* to identify new ways of moving forward and making positive changes for themselves:

*The act of clarifying someone’s difficulties and the process of helping them to feel*

*listened to, understood and accepted is enough to allow them to move forward*

*again (P7)*

*They feel less judged/labelled, and consequently more motivated/hopeful to address*

*issues within treatment (P30)*

*Has helped them to move forward in a positive way (P41)*

##### Basis for Intervention

Thirdly, participants reported that FCF facilitates progress by identifying suitable avenues for intervention and treatment:

*Can usefully change the direction in which a service-user is being managed and the*

*type of intervention being offered (P24)*

*It is particularly useful when deciding which interventions might most effectively*

*meet an individual’s needs (P33)*

*It can also help to focus and direct treatment more effectively (P42)*

This suggests that some OPDP staff find *treatment* and *intervention* suggestions to be helpful. It may therefore be useful to provide more of these suggestions in future alongside those relating to offender *management* (which are typically the primary focus of FCFs within the OPDP presently).

#### Main Service User Outcomes

The fourth theme extracted from these responses represented five participants who provided opinions on whether FCF can influence “ultimate” offender outcomes such as reductions in reoffending. Three of these five participants suggested that FCF *may* be capable of positively impacting these types of outcomes:

*Potentially reducing recall numbers and further offending (P19)*

*Positive outcome for service user (P22)*

*Has helped them to move forward in a positive way with regards to their risk*

*reduction (P41)*

The remaining two participants were more skeptical of any link between FCF and ultimate offender outcomes, referring to the lack of available evidence on this topic:

*I am aware there is limited evidence supporting the use of formulations on treatment*

*outcomes (P13)*

*It remains to be seen through evaluation studies what the behavioural impact of the*

*approach is (P23)*

The uncertainty demonstrated by these participants highlights the importance of empirically examining any link between FCF and these “ultimate” offender outcomes.

#### Barriers to Usefulness and Effectiveness

The final theme extracted from these responses reflected the opinion of participants who suggested that FCF *can* be useful and effective, but only if certain caveats have been met:

*If it is used and updated regularly (P9)*

*I really think the usefulness or effectiveness of the case formulation depends on its*

*quality (P26)*

*I find case formulations which have been developed only from case records and*

*consultation with the Offender Manager as having very limited use . . . I find*

*formulations which have been developed with the service user over a number of*

*sessions as very useful. (P55)*

These comments suggest that OPDP staff have varying ideas about the circumstances under which FCF can be useful or effective.

## Discussion

The present study has uncovered a number of valuable findings. Firstly, although all participants reported being responsible for writing FCFs, most reported that they were not provided with FCF training during their OPDP induction. The amount of FCF training provided to participants was often found to be inconsistent with their needs. Hours of FCF training varied greatly *within*-roles as well as between them, and was not dependent on the complexity of formulations each participant was expected to write. This suggests that the FCF training provided to OPDP staff should be standardized to ensure that all those requiring FCF training are provided with it, and that this training better corresponds with the complexity of FCFs staff members are required to write. Encouragingly, participants who had received most FCF training were also those most likely to report feeling “Very Confident” in their FCF skills and to have received “Excellent” feedback during their last FCF assessment. This suggests that simply improving the quantity of FCF training delivered to staff is likely to produce significant benefits.

Although psychologists are often perceived to be equipped with advanced FCF skills, the results here suggest this might not always be the case. The quantity of FCF training received by psychologists in this study varied greatly (from 0 to 100 hours), and only a quarter of psychologists whose FCFs had been assessed reported having received “Excellent” feedback at their last assessment. Furthermore, less than half of the psychologists in this study reported feeling “Very Confident” in their FCF skills.

Guillemin et al. (2009) states that in order to fulfil professional requirements and to continue to build essential skills and knowledge “healthcare professionals need to adopt a practice of life-long learning” (p. 197). Together, this evidence suggest that psychologists within the OPDP should be provided with regular training to continually update and develop their FCF skills. It is likely that this training would be welcomed by psychologists, as only a quarter of the psychologists surveyed reported feeling “Very Satisfied” with the quantity and quality of their FCF training.

An additional noteworthy finding was that although over half of participants reported having provided FCF training to others, only a third of these participants reported feeling “Very Confident” in their own FCF skills, and only a fifth of these participants reported having received “Excellent” feedback during the last assessment of their FCF skills (some had never been assessed). This indicates that research should concentrate on investigating *who* provides FCF training to others, how they feel about this, and *when* they are deemed capable of providing this training.

### Suggestions for Training Improvement

Results of the thematic analysis highlighted several different ways in which OPDP staff believe FCF training could be improved. The main suggestion was simply that *much more* FCF training should be provided. This indicates that (after completing initial training) a short but regular package of “refresher” training should be provided to ensure that staff can continue to develop their FCF skills throughout their time working within the OPDP.

Results also revealed a number of ways in which the content and methods of FCF training could be further improved. Staff felt that FCF training should be much more interactive and did not find fictitious case vignettes to be realistic enough. This insight is important, as the literature supports the idea of using a mixture of passive and active learning methods to maximize learning outcomes; first through watching/listening, and then through doing/interacting (i.e., MacDonald & Frank, 2016; Minhas et al., 2012). By enabling staff to *actively* apply their newly learned skills to genuine and complex cases, they would be better equipped to deal with these types of cases in practice. It is possible that using genuine case information could present some ethical challenges (i.e., relating to the anonymity of case information), however, the value of using this method suggests that it would be worth exploring ways of overcoming these challenges, such as asking for prior consent from offenders before using their case information within training.

Staff also felt that FCF training should include much more psychological content. This is imperative, as one of the main purposes of formulation within the OPDP is to develop a *psychological* understanding of the causes and development of each service user’s presenting problems. However, as earlier discussed, OMs often have less psychological knowledge and training than psychologists (although they are expected to write formulations within the OPDP). It therefore must be ensured that in future, OMs come away from OPDP formulation training with the necessary skills to formulate complex cases in practice. To do this, FCF training must also consider individual staff needs to ensure it is accessible for all.

These findings suggest that with the implementation of some relatively small changes, staff are likely to feel much more satisfied with the quantity and quality of their FCF training. Any such changes should be first discussed with OPDP staff to determine how best to incorporate these, as the benefits of co-design have been shown to include higher user satisfaction and a better fit between the needs of the user and the service provided (Steen et al., 2011).

### Opinions on the Usefulness and Effectiveness of Formulation

Thematic analysis revealed that all participants considered FCF to be useful and effective in at least one way. Staff indicated that FCF can improve staff understanding of and empathy toward offenders within the OPDP. This finding complements previous studies which have identified that forensic case consultation (the first step in producing a FCF) can improve a range of self-reported staff outcomes such as understanding, empathy, insight, and awareness toward offenders (Knauer et al., 2017; McMullan et al., 2014; Ramsden et al., 2014; Whitton et al., 2016). It may also be possible for these increases in staff understanding and empathy to lead to further positive improvements; Shaw et al (2017) found that OMs who completed FCFs collaboratively with offenders later reported significantly higher-quality relationships with these service users than OMs who formulated as normal (non-collaboratively). Although not specifically investigated by Shaw et al. (2017), it is feasible that constructing FCFs collaboratively with offenders increased staff understanding and empathy toward them, resulting in the improved relationships reported.

Supporting this theory, some participants within the current study did report that FCF was capable of improving relationships between staff and offenders. This is an important finding when placed in the context of research by Skeem et al. (2007), who identified that the strength of probation officer-offender relationships in the USA could predict recidivism over an average follow-up period of 16 months. It would therefore be valuable to investigate whether stronger OPDP staff-offender relationships (as facilitated by FCF) may also be associated with positive offender outcomes such as reduced reoffending risk or improved well-being. This type of research is likely to produce a more comprehensive understanding of the true utility and value of FCF within the OPDP.

Staff also indicated that FCF is useful for providing new perspectives and approaches to take when working with difficult or “stuck cases.” This suggests that FCF may act as a catalyst; first producing smaller and more direct impacts (i.e., improving staff understanding of offenders and identifying new directions), which in turn can lead to further positive impacts (improved relationships, improved engagement), potentially resulting in “ultimate” positive outcomes such as reductions in reoffending.

The theory that FCF is the first “step” in a much larger process may explain why only a very small number of survey participants directly commented on the ability of FCF to influence these “ultimate” service user outcomes (such as reductions in reoffending). The mixed nature of these comments also corresponds with previous research by Völlm (2014), who found that only 40% of formulation experts agreed with the statement that offenders who receive a FCF are more likely to achieve a positive case outcome. Together, these findings suggest that until rigorous study is conducted to measure the potential impact of FCF on “ultimate” outcomes, this is likely to remain a hotly debated topic within the FCF field.

Finally, many participants indicated that the usefulness and effectiveness of FCF is dependent on certain factors (such as whether it is of high quality, whether it has been constructed collaboratively with the service user, and/or whether it has been updated over time). This may be due to differing experiences, or because it is not yet known which FCFs are objectively likely to be most useful or effective (as no validated measure of formulation quality yet exists). This finding suggests that research should aim to clarify both *if* and *when* (i.e., under which circumstances) FCF can have a positive impact within the OPDP.

### Study Limitations

Although a relatively small number of participants took part in this survey (*N* = 55), those who did take part worked within a range of different OPDP teams across the UK, held a range of different job roles, and had a variety of different FCF training experiences. However, it is recognized that a sample of 55 participants is only small proportion of the OPDP workforce, and so the conclusions made on the basis of this study should be regarded with that in mind.

Secondly, although the survey did allow for a wide range of both quantitative and qualitative data to be collected, face to face interviews may have resulted in richer data. Although this was unfortunately not feasible in this instance, the majority of participants did provide detailed responses to the survey, and their opinions and experiences should be used when shaping any future FCF training developed for use within the OPDP to better ensure it is of the highest quality and can best meet the needs of staff.

To further confirm the findings, a quantitative study should be conducted to prove statistically whether the quantity and/or quality of formulation training received by OMs and psychologists has any noticeable impact on the quality of formulations they are able to produce. However, this should not delay the process of improving the quality and quantity of formulation training in the OPDP currently.

## Conclusion

This study has highlighted that there is currently no standardized amount, frequency, or source of FCF training provided to OPDP staff. Staff satisfaction with FCF training may be rapidly improved by offering additional and/or refresher FCF training, which is also likely to improve confidence and capability in writing high-quality FCFs. Any such training should be developed in line with the recommendations provided here by staff in order to maximize its quality and accessibility.

## Notes

1. Aside from the service manager, who reported never having received formulation training.

2. “Kahootz” is an information sharing platform used by OPDP staff.
